# Thermal requirement of indian mustard (*Brassica juncea*) at different phonological stages under late sown condition

**DOI:** 10.1007/s40502-014-0072-0

**Published:** 2014-09-03

**Authors:** Manoj Pratap Singh, Lallu, N. B. Singh

**Affiliations:** 1 Department of Crop Physiology, C.S.A. University of Agriculture and Technology, Kanpur, 208002 Uttar Pradesh India; 2 Section of Rabi Cereal, C.S.A. University of Agriculture and Technology, Kanpur, 208002 Uttar Pradesh India

**Keywords:** Growing degree days, Heat use efficiency, Helios thermal unit, Photo-thermal index, Photo-thermal unit

## Abstract

Indian mustard [*Brassica juncea* (L.) Czern & Coss.] is a long day plant, which requires fairly cool climatic condition during growth and development for obtaining better seed yield. Various workers have correlated crop growth and development with energy requirement parameters, such as growing degree days (GDD), photo-thermal unit (PTU), helios thermal unit (HTU), photo-thermal index (PTI) and heat use efficiency (HUE). Therefore, GDD requirement for different phenological stages of 22 newly developed Indian mustard varieties was studies during winter (*rabi*) seasons of 2009–10 and 2010–11 at student instructional farm of C.S. Azad University of Agriculture and Technology, Kanpur, (Utter Pradesh). Study revealed that RH-8814, NRCDR-02 and BPR-549-9 recorded higher GDD (1703.0, 1662.9 and 1648.0), PTU (19129.8, 18694.2 and 18379.8), HTU (11397.7, 11072.2 and 10876.0), PTI (13.25, 13.14 and 13.08) and HUE (4.11, 3.84 and 3.71) at physiological maturity, while higher HUE was recorded (9.62, 8.99 and 8.91 kg ha^−1^ degrees-day) at days after sowing (DAS) to 50 % flowering. On the basis of study mustard genotypes RH-8814, NRCDR-02 and BPR-549-9 were identified as most heat-tolerant, as they maintained higher values for energy related parameters. Seed yield was highly positively correlated with GDD (r = 0.61, 0.65 and 0.75), PTU (r = 0.66, 0.64 and 0.74), HTU(r = 0.79, 0.68 and 0.73) at the above these three phenological stages, while negatively correlated with PTI at anthesis and at maturity. Hence, these parents could be used in crossing programme for achieving further improvement.

## Introduction

Indian mustard [*Brassica juncea* (L.) Czern & Coss.] is a crop of tropical as well as temperate zones requiring somewhat cool and dry weather for satisfactory growth and development. High temperature at flowering stage causes reduction in seed yield as it may lead to pollen sterility. Although mustard is a long day plant requiring 16 h of light period in 24 h cycle, it can made to flowering if it is provided with a cycle of 8 h of light period with 4 h of dark period (short night). Mustard can be made to flower in about 50 days under 16/8 h light/dark period.

Growing degree days (GDD), photo-thermal unit (PTU), helios thermal unit (HTU), photo-thermal index (PTI) and heat use efficiency (HUE) have frequently been used as a weather based parameters for assessing crop phenology. Therefore, all growth and development stages of crop may be estimated more accurately on the basis of GDD rather than calendar method (Warthington and Hatchinson 2005). Mustard crop require different amount of GDD, PTU, HTU, PTI and HUE for growth and development stages. The GDD is used to quantify effect of temperature and described the timing of different biological process (McMaster and Wilhelm 1997; Qiao-yan et al. 2012). The present investigation was carried out to quantify relationship of GDD, PTU, HTU, PTI and HUE with phenological development of crop.

## Materials and methods

Field experiments were conducted during the winter (*Rabi*) seasons of 2009–10 and 2010–11 at student instructional farm of C.S. Azad University of Agriculture & Technology, Kanpur, Utter Pradesh, India, with the objective to study the relationship between GDD, PTU, HTU, PTI and HUE at different phenological stages with seed yield, and thereby to evaluate twenty two Indian mustard genotypes for high temperature tolerance under late sown condition. The experiment site situated at a height 125.9 m above mean sea level at 26^°^39′N latitude and 80^°^18′E longitude had a semi-arid type of climate. The soil of the experimental field was sandy loam in texture having 0.45 % organic carbon, 267.0 kg ha^−1^ available N, 18.2 kg ha^−1^ available P and 168.5 kg ha^−1^ available K with pH 7.4. Average temperature during crop grown season was 20.5 ± 1 °C. Experiment were laid out in randomize block design with three replications. The weather conditions (Table 1) (climatic features) in respect of rainfall, temperature, relative humidity and sun shine hours of the relevant years were obtained from the Agronomy Department of the C.S. Azad university of Agriculture and Technology, Kanpur.

**Table 1:** Weather data of standard meteorological week’s from November to April, 2009–10 and 2010–11 at C.S. Azad university of Agriculture and Technology, Kanpur (*T* Temperature, *RH* relative humidity, *RF* rainfall, *BSS* bright sun shine h)

Meteorological weeks	2009–10						2010–11					
	T (°C)		RH (%)		RF day^−1^ (mm)	BSS (h)	T (°C)		RH (%)		RF day^−1^ (mm)	BSS (h)
	Max.	Min.	Max.	Min.			Max.	Min.	Max.	Min.		
45	30.66	13.63	90.14	38.86	00.0	7.5	29.96	17.66	90.14	48.29	0.0	3.0
46	27.27	16.90	87.00	58.43	1.5	3.9	26.40	17.06	91.29	73.57	8.1	3.5
47	24.84	12.56	82.71	47.57	00.0	3.8	26.23	16.74	95.57	69.14	2.5	3.5
48	26.17	8.94	85.86	31.86	00.0	3.4	24.17	9.29	86.43	66.00	1.5	7.8
49	25.54	8.90	91.43	41.86	00.0	5.6	24.63	11.40	83.86	50.86	0.0	6.1
50	25.69	10.74	90.00	41.43	00.0	3.5	23.54	7.73	85.29	35.29	0.0	5.6
51	24.14	10.57	89.29	43.43	00.0	3.7	23.60	7.31	84.86	35.29	0.0	5.9
52	22.10	6.70	89.57	41.29	00.0	5.6	23.06	9.64	91.71	48.29	0.2	2.4
53	17.36	8.03	94.29	69.57	1.4	2.4	–	–	–	–	–	–
1	16.04	7.56	95.57	81.71	00.0	0.5	11.73	4.23	95.71	74.57	0.0	0.2
2	14.09	8.54	95.57	80.00	0.5	0.5	20.51	5.29	89.43	48.71	0.0	3.4
3	15.94	6.63	93.57	71.57	00.0	1.6	22.80	6.80	84.00	32.71	0.0	7.0
4	24.11	8.03	91.57	46.14	00.0	6.1	23.14	8.06	86.43	49.86	0.0	6.2
5	25.14	9.29	90.57	39.29	00.0	7.0	25.23	9.71	90.57	44.14	0.0	6.2
6	23.10	13.71	95.14	69.29	1.6	3.3	26.56	11.91	85.57	38.71	0.0	8.8
7	25.31	10.97	85.00	54.86	00.0	7.0	23.40	11.69	92.00	55.00	0.9	4.6
8	28.94	13.90	86.14	43.86	00.0	8.6	25.17	11.61	89.43	45.71	0.3	6.6
9	31.86	16.26	80.14	36.57	00.0	8.0	28.09	13.39	77.14	38.00	0.0	8.1
10	30.83	14.19	76.71	29.71	00.0	9.7	29.51	14.91	73.57	36.29	0.0	9.8
11	34.23	17.37	80.14	28.00	00.0	8.7	33.51	16.63	77.57	43.14	0.0	8.5
12	39.14	19.10	74.14	20.71	00.0	8.3	34.86	16.71	72.57	47.29	0.0	9.5
13	38.46	19.63	57.71	24.14	00.0	8.2	33.77	19.17	82.43	58.29	0.0	8.1
14	40.14	20.96	32.14	17.43	00.0	8.3	36.23	16.89	55.71	27.71	0.1	8.5
15	42.54	22.46	35.43	13.14	00.0	9.7	37.37	19.63	50.00	21.29	0.4	6.7
16	41.46	28.20	38.43	15.29	00.0	5.83	37.23	20.80	59.86	22.57	1.1	8.8
17	41.44	34.20	60.00	22.40	00.0	7.64	37.80	23.60	56.00	28.29	0.0	8.0

All energy requirement parameters were recorded for three durations, viz., sowing to anthesis, sowing to 50 % flowering and sowing to physiological maturity.

Growing degree days was computed with 5 °C as base temperature on the basis of daily mean temperature with the help of following formula:

$$ GDD = \frac{{\sum (Tem.{\hbox{max}} - {Tem}.\hbox{min} )}}{2} - Tb $$

Where, *Tem.max* and *Tem.min* are daily maximum and minimum temperature, Tb is the base temperature (5 °C).

Photo-thermal unit (PTU) (degree-days hours) was calculated on the basis of GDD and day length with the formula given below:

$$ \text{PTU} = GDD \times Day \, length $$

Helios thermal unit (HTU) (degree-days hours) was calculated on the basis of GDD and sunshine hours by the following formula:

$$ {\text{HTU}} = {\text{GDD}} \times {\text{Duration of sunshine hours}} $$

Photo-thermal index (PTI) (degree-days day^−1^) was calculated using the following equation:

$$ \text{PTI} = GDD/Growth \, days $$

Heat use efficiency (HUE) (kg ha^−1 ^degrees-day) was calculated with the help of seed yield (kg^−1^ha) per GDD with the help of following equation:

$$ {\text{HUE}} = {\text{Seed yield}}\left( {{\text{kg ha}}^{ - 1} } \right)/{\text{GDD}} $$

## Results and discussion

Growing degree days (GDD) (degree-days) calculated for durations of sowing to anthesis, sowing to 50 % flowering and sowing to physiological maturity showed significant differences among tested mustard genotypes under late sown situation (Table 2). Pooled data analysis of both the years indicated higher values of GDD at anthesis, 50 % flowering and at physiological maturity in RH-8814 (595.1, 726.6 and 1703.0) followed by NRCDR-02 (573.6, 713.5 and 1669.2) and in BPR-549-9 (572.0, 685.8 and 1648.0), while the lowest GDD values were recorded in NPJ-113 (445.2, 543.5 and 1351.5) and in NPJ-117 (450.4, 559.0 and 1391.8) under late sown condition. Results on GDD at various phonological stages are in conformity of results reported by Renganayaki and Krishnasamy (2013).

**Table 2:** Growing degree days, photo-thermal unit and helios thermal unit at different growth stages in Indian mustard under late sown condition (pooled 2 years)

Genotypes	Traits								
	GDD (degree-days)			PTU (degree-days hour)			HTU (degree-days hour)		
	Days to anthesis	Days to 50 % flowering	Days to Physiological maturity	Days to anthesis	Days to 50 % flowering	Days to physiological maturity	Days to anthesis	Days to 50 % flowering	Days to physiological maturity
DRMR-802	540.3	662.2	1,569.2	5,555.7	6,897.5	17,497.8	2,338.1	3,036.2	10,276.2
PBR-331	527.7	632.1	1,533.6	5,417.7	6,565.5	16,946.4	2,266.9	2,834.6	9,928.4
NDC-601	533.2	650.1	1,512.1	5,479.2	6,625.2	16,822.8	2,233.6	2,935.5	9,848.8
RH-0,216	504.9	594.0	1,453.9	5,166.9	6,146.4	15,990.9	2,205.4	2,615.9	9,218.0
NPJ-113	445.2	543.5	1,351.5	4,535.2	5,591.5	14,761.5	2,084.3	2,407.2	8,281.7
RB-50	527.1	637.8	1,510.7	5,410.5	6,628.2	16,672.5	2,225.9	2,865.4	9,737.5
NPJ-117	450.4	559.0	1,391.8	4,587.4	5,762.0	15,245.1	2,084.3	2,485.1	8,656.3
RH-0,447	496.4	615.8	1,432.4	5,215.3	6,386.2	15,732.3	2,212.5	2,738.7	9,014.6
DLM-2	522.4	650.1	1,567.6	5,358.8	6,625.2	17,354.4	2,289.8	2,935.5	10,202.1
RH-0,305	523.1	605.8	1,402.2	5,224.6	6,276.2	15,609.9	2,181.6	2,681.2	8,934.0
NRCDR-701	524.6	649.1	1,521.7	5,453.4	6,753.4	16,803.9	2,353.3	2,949.1	9,825.1
RH-0,116	524.4	662.4	1,535.1	5,381.4	6,899.1	16,965.0	2,266.9	3,026.8	9,948.3
NRCDR-02	573.6	713.5	1,669.2	5,992.4	7,460.9	18,694.2	2,556.5	3,344.5	11,072.2
RB-55	543.8	643.6	1,589.6	5,594.8	6,692.3	17,618.4	2,342.4	2,897.5	10,344.6
RH-8,814	595.1	726.6	1,703.0	6,228.4	7,605.6	19,129.8	2,643.3	3,448.7	11,397.7
DRMR-537-40	536.2	650.1	1,569.2	5,511.2	6,625.2	17,373.6	2,303.3	2,935.5	10,205.8
RGN-773	548.4	674.3	1,635.9	5,644.8	7,029.7	18,234.0	2,371.0	3,130.2	10,783.0
PBR-330	546.4	640.0	1,600.5	5,624.4	6,652.4	17,779.2	2,345.8	2,904.1	10,462.5
RGN-197	522.0	627.3	1,565.8	5,355.0	6,513.0	17,332.8	2,266.9	2,801.6	10,171.0
BPR-349-9	557.0	655.7	1,600.5	5,739.4	6,686.3	17,779.2	2,461.2	2,987.1	10,462.5
BPR-549-9	572.0	685.8	1,648.0	5,904.4	7,156.8	18,379.8	2,492.7	3,209.7	10,876.0
RH-555A	529.0	655.5	1,577.6	5,431.4	6,822.9	17,474.9	2,303.3	3,000.9	10,270.5
Mean	529.2	642.4	1,542.7	5,446.0	6,654.6	17,099.9	2,310.4	2,916.9	9,742.2
SE (d) ±	10.2	8.6	3.9	21.5	96.4	156.1	27.4	48.5	62.5
CD at 5 %	20.6	17.3	8.0	43.3	194.3	315.1	55.2	97.7	126.1

Photo-thermal unit (PTU) (degree-days hours) increased linearly with increase in plant age up to physiological maturity of crop in both the years of study. PTU recorded at anthesis, 50 % flowering and at physiological maturity ranged from 4961.7 to 6488.2, 5851.3 to 7619.0 degree-days hours. Based on pooled analysis the highest value of PTU was observed in RH-8814 (6228.4, 7605.6 and 19129.8), followed by NRCDR-02 (5992.4, 7460.9 and 18694.2) and in BPR-549-9 (5904.4, 7156.8 and 18379.8), while the lowest PTU were recorded in NPJ-113 (4535.2, 5591.5 and 14761.5) and NPJ-117 (4587.4, 5762.0 and 15245.1) at anthesis, 50 % flowering and physiological maturity under late sown condition. Khushu et al. (2008) reported that the photo-thermal units (PTU) were highest in early sowing (D_1_), followed by late sowings (D_2_ and D_3_).

Helios thermal unit (HTU) (degree-days hours) recorded at anthesis, 50 % flowering and at physiological maturity (Table 2) increased linearly with increase in duration of crop during both the years of investigation, and showed significant variability among 22 mustard genotypes. Based on pooled analysis, at anthesis, 50 % flowering and at physiological maturity, the higher values of HTU were recorded in RH-8814 (2643.3, 3448.7 and 11397.7), followed by NRCDR-02 (2556.5, 3344.5 and 11072.2) and BPR-549-9 (2492.7, 32.09.7 and 10876.0), while the lower values of HTU were recorded in NPJ-113 (2084.3, 2407.2 and 8281.7), followed by NPJ-117 (2084.3, 2485.1 and 8656.3) under late sown condition. These result are in conformity of the results reported on phenological development in mustard crop by Srivastava and Balkrishna (2003).

Results of PTI (degree-days day^−1^) at anthesis revealed highest values in genotypes NPJ-113 (10.60), followed by NPJ-117 (10.47) and RH-447 (10.03) (Table 3). However, at 50 % flowering and physiological maturity the higher values for PTI were observed in genotypes RH-8814 (10.02 and 13.25), NRCDR-02 (9.98 and 13.14) and BPR-549-9 (9.87 and 13.08). Similar results on PTI were supported by Prasanta Neog Chakravarty (2005).

**Table 3:** Photo-thermal index, heat use efficiency and seed yield plant^−1^ in Indian mustard under late sown condition (Pooled 2 years)

Genotypes	Traits						
	Photo-thermal index (degree-days day^−1^)			Heat use efficiency (kg ha^−1^ degrees-day)			Seed yield (g plant^−1^)
	Days to anthesis	Days to 50 % flowering	Days to physiological maturity	Days to anthesis	Days to 50 % flowering	Days to physiological maturity	
RH-0305	9.87	9.69	12.30	0.62	5.34	2.30	20.20
RB-50	9.85	9.74	12.59	0.72	5.92	2.50	23.60
RH-0447	10.03	9.70	12.30	0.83	6.65	2.86	25.60
RGN-773	9.79	9.84	13.04	0.85	6.95	2.87	29.30
RH-0116	9.89	9.81	12.69	0.89	7.03	3.03	29.10
RH-555A	9.89	9.78	12.83	0.91	7.35	3.05	30.10
BPR-349-9	9.86	9.79	12.91	0.93	7.88	3.23	32.30
PBR-330	9.76	9.77	12.91	0.95	8.10	3.24	32.40
DRMR-802	9.82	9.81	12.81	0.94	7.71	3.25	31.90
DLM-2	9.95	9.78	12.80	0.98	7.90	3.28	32.10
NPJ-117	10.47	9.89	12.16	1.02	8.19	3.29	28.60
RH-0216	10.00	9.74	12.37	0.96	8.13	3.32	30.20
NDC-601	9.78	9.78	12.60	0.95	7.75	3.33	31.50
RGN-197	9.67	9.73	12.78	1.01	8.42	3.37	33.00
PBR-331	9.86	9.72	12.67	0.99	8.25	3.40	32.60
NPJ-113	10.60	9.88	12.01	1.04	8.54	3.43	29.00
DRMR-537-40	9.84	9.78	12.81	1.03	8.49	3.52	34.50
NRCDR-701	9.81	9.76	12.63	1.04	8.38	3.57	34.00
RB-55	9.80	9.75	12.87	1.06	8.92	3.61	35.90
BPR-549-9	9.78	9.87	13.08	1.07	8.91	3.71	38.20
NRCDR-02	9.64	9.98	13.14	1.12	8.99	3.84	40.10
RH-8814	9.60	10.02	13.25	1.17	9.62	4.11	43.70
Mean	9.87	9.81	12.72	0.96	7.90	3.29	31.72
SE (d) ±	0. 11	0.11	0.11	0.09	0.10	0.09	0.16
CD at 5 %	0.22	0.22	0.22	0.18	0.21	0.18	0.32

Heat use efficiency (HUE) (kg ha^−1 ^degrees-day) recorded at anthesis, 50 % flowering and physiological maturity (Table 3 showed higher values for genotypes RH-8814 (1.17, 9.62 and 4.11), NRCDR-02 (1.12, 8.99 and 3.84) and BPR-549-9 (1.07, 8.91 and 3.74) at anthesis, 50 % flowering and physiological maturity, respectively. Results on HUE are in conformity of the results reported by Prasanta Neog Chakravarty (2005).

Seed yield plant^−1^ recorded at harvest (Table 3) showed significant genotypic variation among 22 genotypes under late sown condition during 2009–10 and 2010–11. Crop faces high temperature under late sown condition at seed development phase. Pooled data of two crop seasons (Table 4) revealed that seed yield per plant was highest in RH-8814 (43.7 g), NRCDR-02 (40.10 g), and BPR-549-9 (38.20 g). These finding are in conformity with the results reported by Singh et al. (2001) and (2013). Studies of correlation matrix of seed yield with GDD (r = 0.61, 0.65 and 0.75), PTU (r = 0.66, 0.64 and 0.74), HTU (r = 0.79, 0.68 and 0.73) were highly significant at anthesis, 50 % flowering and at physiological maturity (Table 4). From the data it is evident that GDD, PTU, HTU, PTI and HUE can be recommended as indices for selection of genotypes for high temperature tolerance. Further, genotypes RH-8814, NRCDR-02 and BPR-549-9 having highest GDD, PTU, HTU, PTI and HUE as well as seed yield can be recommended for breeding programme for developing high yielding genotypes for late sown-high temperature condition.

**Table 4:** Correlation of growing degree days, photo-thermal unit, helios thermal unit, photo-thermal index and heat use efficiency with seed yield of mustard genotypes under late sown condition

Traits	2	3	4	5	6	7	8	9	10	11	12	13	14	15	16
Seed yield (1)	0.61**	0.65**	0.75**	0.66**	0.64**	0.74**	0.79**	0.68**	0.73**	−0.01	0.05	−0.17	0.15	0.15	0.18*
GDD at anthesis (2)	–	0.94**	0.91**	0.99**	0.93**	0.93**	0.92**	0.92**	0.93**	−0.08	0.01	−0.07	0.24**	0.24**	0.27**
GDD at 50 % flowering (3)		–	0.92**	0.96**	0.99**	0.93**	0.91**	0.99**	0.94**	−0.03	0.01	−0.06	0.21*	0.23**	0.26**
GDD at maturity (4)			–	0.92**	0.92**	0.99**	0.15	0.18*	0.26**	−0.14	0.07	−0.03	0.31**	0.33**	0.37**
PTU at anthesis (5)				–	0.95**	0.94**	0.94**	0.94**	0.94**	−0.08	0.01	−0.08	0.24**	0.24**	0.24**
PTU at 50 % flowering (6)					–	0.93**	0.91**	0.99**	0.93**	−0.07	0.00	−0.03	0.21*	0.22*	0.26**
PTU at maturity (7)						–	0.92**	0.94**	0.99**	−0.14	0.05	−0.03	0.30**	0.31**	0.36**
HTU at anthesis (8)							–	0.93**	0.92**	−0.11	0.10	−0.01	0.30**	0.30**	0.34**
HTU at 50 % flowering (9)								–	0.94**	−0.05	0.03	−0.13	0.23**	0.24**	0.28**
HTU at maturity (10)									–	−0.13	0.05	−0.05	0.29**	0.31**	0.35**
PTI at anthesis (11)										–	0.01	−0.82	−0.06	−0.09	−0.18
PTI at 50 % flowering (12)											–	0.40**	0.63**	0.58**	0.63**
PTI at maturity (13)												–	0.39**	0.40**	0.46**
HUE at anthesis (14)													–	0.99**	0.98**
HUE at 50 % flowering (15)														–	0.98**
HUE at maturity (16)															1.00

*, ** significant 0.05 and 0.01 levels
